# Supplementary material for: Rhythmic Clock Gene Expression in Atlantic Salmon Parr Brain
Source: Front Physiol. 2021 Dec 2;12:761109. doi: 10.3389/fphys.2021.761109 (PMC8674837; doi:10.3389/fphys.2021.761109)
Supplement: Supplementary file 3 [file Data_Sheet_3.PDF]

# Circadian clocks in salmonids - the effect of the genome duplication on clock genes

## SUPPLEMENTARY MATERIAL

### Supplementary Figure S1

Phylogenetic relationship of the *period* gene family. Values on the relevant node depict the bootstrap values. Sequence ID used and identified are provided.

### Supplementary Figure S2

Phylogenetic relationship of the *clock* gene family. Values on the relevant node depict the bootstrap values. Sequence ID used and identified are provided.

### Supplementary Figure S3

Phylogenetic relationship of the *aryl hydrocarbon receptor nuclear translocator-like* gene family (*arntl*). Values on the relevant node depict the bootstrap values. Sequence ID used and identified are provided.

### Supplementary Figure S4

Phylogenetic relationship of the *cryptochrome* gene family (*cry*). Values on the relevant node depict the bootstrap values. Sequence ID used and identified are provided.

### Supplementary Figure S5

Phylogenetic relationship of the *nuclear receptor subfamily 1 group d* gene family (*nr1d*). Values on the relevant node depict the bootstrap values. Sequence ID used and identified are provided.

### Supplementary Figure S6

Phylogenetic relationship of the *RAR-reticulated orphan receptor* (*ror*). Values on the relevant node depict the bootstrap values. Sequence ID used and identified are provided.

### Supplementary Figure S7

Phylogenetic relationship of the *casein kinase 1 delta* (*csnk1d*) and *epsilon* (*csnk1e)*. Values on the relevant node depict the bootstrap values. Sequence ID used and identified are provided.

### Supplementary Figure S8

Significantly cyclical gene expression. Parameters of the cyclic sin-cosine function calculated by MetaCycle with JTK ($P < 0.001$) for diel expression of clock genes in brains collected from Atlantic salmon smolt exposed to an LD 12:12 photoperiod.

### Supplementary Figure S9

Heatmap displaying individual diel expression of identified clock genes under constant LD (12:12, n = 6 per time point). The heatmap of the relative expression of each individual gene [scaled from lowest expression to highest expression]. Black diamond indicates significantly cyclic gene ($p < 0.001$) [JTK and RAIN analysis], White circles denote rhythmic genes ($p < 0.001$) [RAIN analysis].

### Supplementary Table S1

Samples and reads details.

### Supplementary Table S2

*S. salar* clock gene accessions and locations.

**Supplementary Table S3**

Number of significant genes: Rhythmic (RAIN) or circadian (JTK) depending on the *P*-value (FDR adjusted) or relative Amplitude thresholds used. All dataset and clock genes only (in bracket).

**Supplementary Data S1**

The expression level. For each sample, estimate gene abundance expressed in the fragments per kilobase of exon per million mapped reads (FRKM) as exported by StringTie2/HiSat2. (CSV)

**Supplementary Data S2**

All rhythmically expressed genes (after Transcripts Per Million (TPM) scaling and normalisation). (CSV)

## SUPPLEMENTARY FIGURES

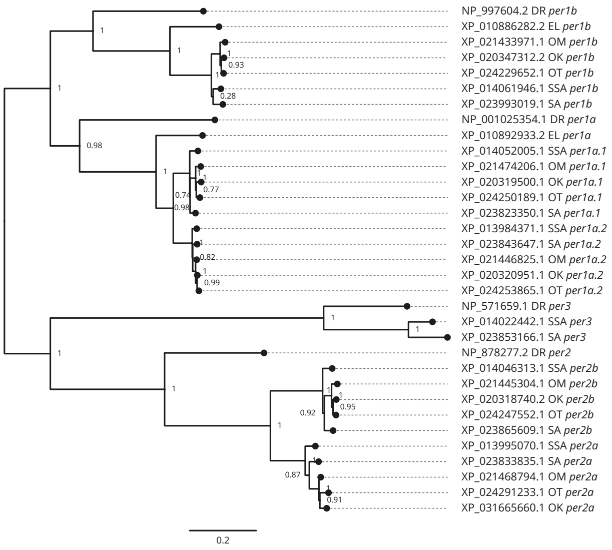


**Supplementary Figure S1.** Phylogenetic relationship of the *period* gene family. Values on the relevant node depict the bootstrap values. Sequence ID used and identified are provided.

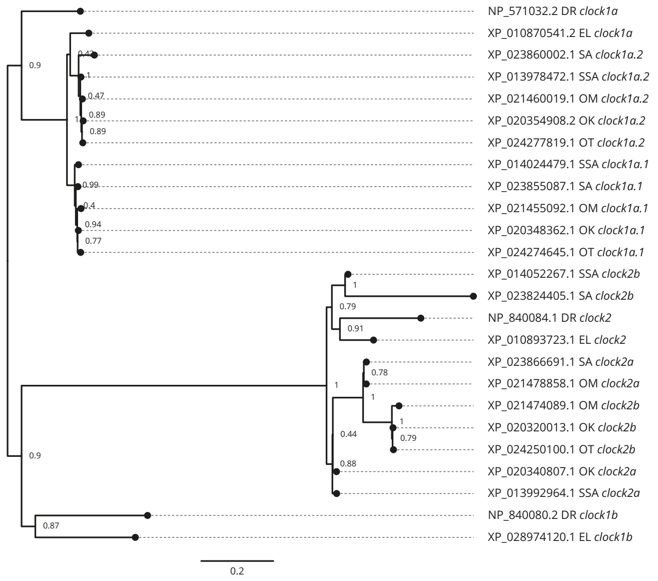


**Supplementary Figure S2.** Phylogenetic relationship of the *clock* gene family. Values on the relevant node depict the bootstrap values. Sequence ID used and identified are provided.

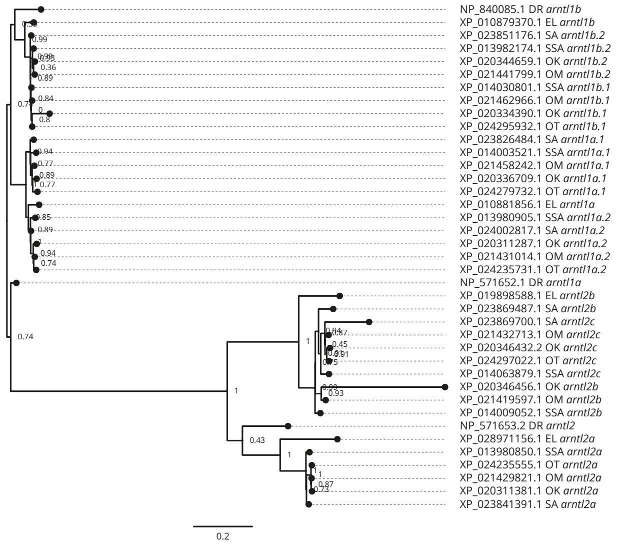


**Supplementary Figure S3.** Phylogenetic relationship of the *aryl hydrocarbon receptor nuclear translocator-like* gene family (*arntl*). Values on the relevant node depict the bootstrap values. Sequence ID used and identified are provided.

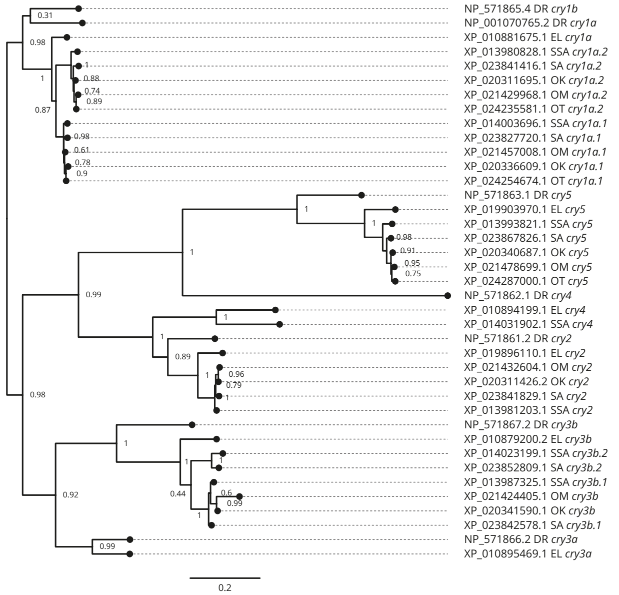


**Supplementary Figure S4.** Phylogenetic relationship of the *cryptochrome* gene family (*cry*). Values on the relevant node depict the bootstrap values. Sequence ID used and identified are provided.

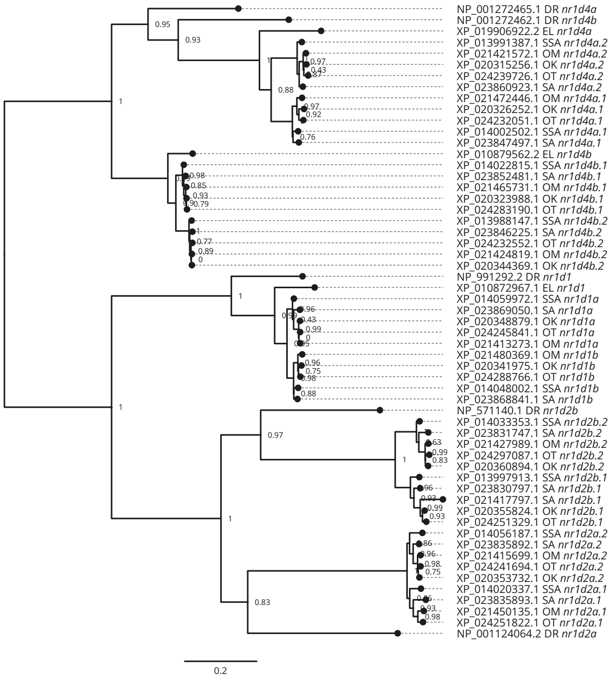


**Supplementary Figure S5.** Phylogenetic relationship of the *nuclear receptor subfamily 1 group d* gene family (*nr1d*). Values on the relevant node depict the bootstrap values. Sequence ID used and identified are provided.

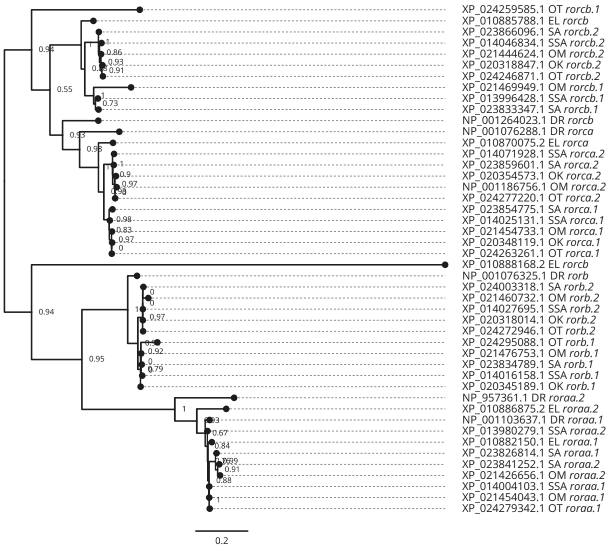


**Supplementary Figure S6.** Phylogenetic relationship of the *RAR-reticulated orphan receptor* (*ror*). Values on the relevant node depict the bootstrap values. Sequence ID used and identified are provided.

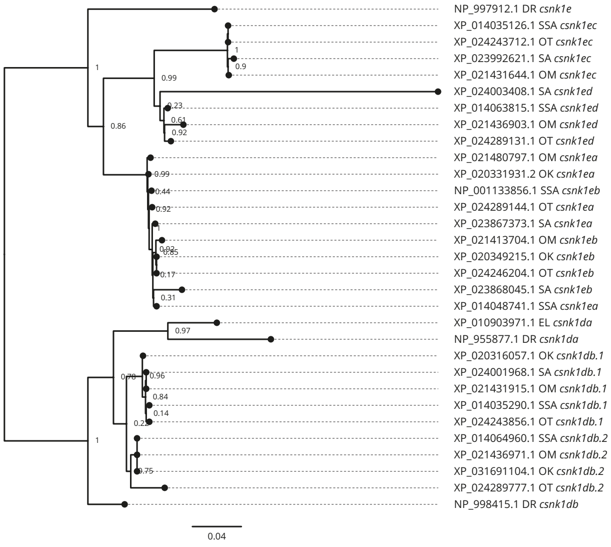


**Supplementary Figure S7.** Phylogenetic relationship of the *casein kinase 1 delta* (*csnk1d*) and *epsilon* (*csnk1e*). Values on the relevant node depict the bootstrap values. Sequence ID used and identified are provided.

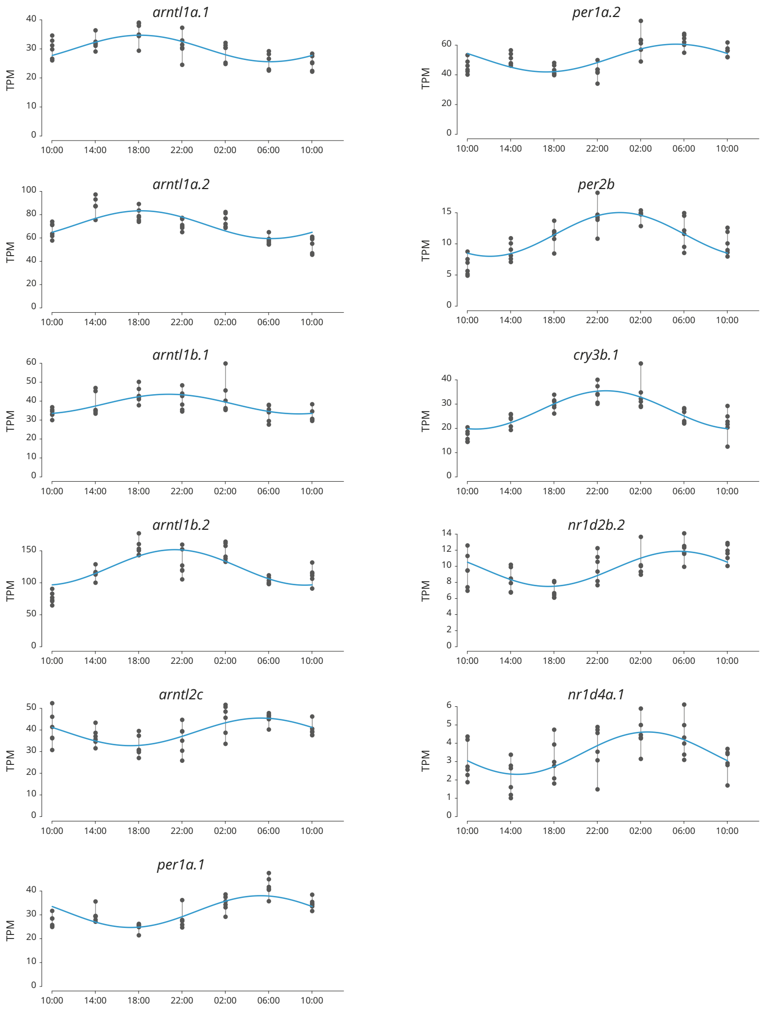


**Supplementary Figure S8.** Significantly cyclical gene expression. Parameters of the cyclic sin-cosine function calculated by MetaCycle with JTK ($P < 0.001$) for diel expression of clock genes in brains collected from Atlantic salmon smolt exposed to an LD 12:12 photoperiod.

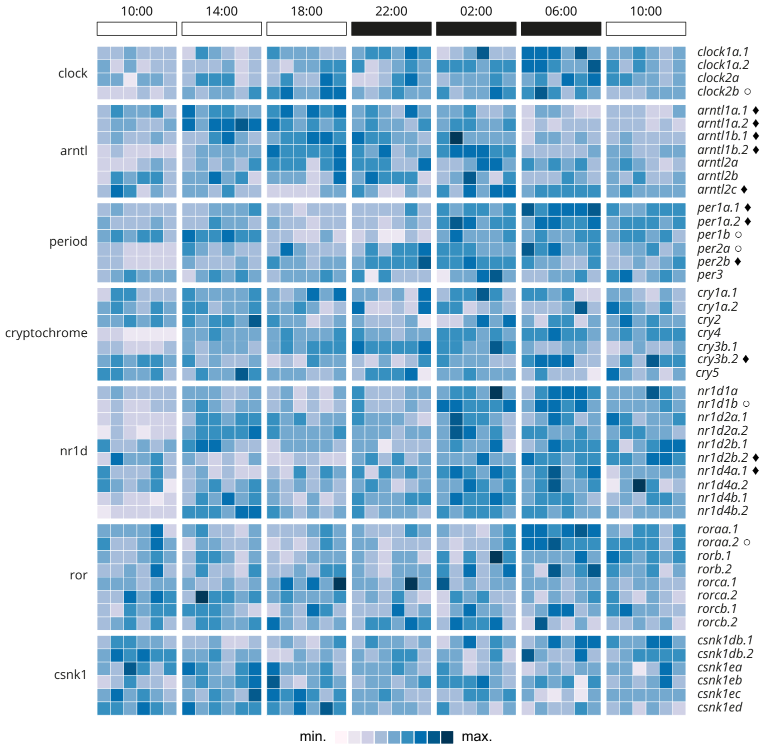


**Supplementary Figure S9.** Heatmap displaying individual diel expression of identified clock genes under constant LD (12:12, n = 6 per time point). The heatmap of the relative expression of each individual gene [scaled from lowest expression to highest expression]. Black diamond indicates significantly cyclic gene ($p < 0.001$) [JTK and RAIN analysis], White circles denote rhythmic genes ($p < 0.001$) [RAIN analysis].

## SUPPLEMENTARY TABLES

**Supplementary Table S1.** Samples and reads details.

| Sample | SampleID | BrainID | Sample EBI Accession | Raw Reads | Date | Time (ZT) | Timepoint | Mapping |
|---|---|---|---|---|---|---|---|---|
| A1 | 2/LD/T0/1 | 85 | ERS5329244 | 47723702 | 2020-08-24T09:00:00Z | 0 | 10:00 | 97.8% |
| A2 | 2/LD/T0/2 | 86 | ERS5329245 | 41776194 | 2020-08-24T09:00:00Z | 0 | 10:00 | 98.0% |
| A3 | 2/LD/T0/3 | 87 | ERS5329246 | 48551618 | 2020-08-24T09:00:00Z | 0 | 10:00 | 97.9% |
| A4 | 2/LD/T0/4 | 88 | ERS5329247 | 40458840 | 2020-08-24T09:00:00Z | 0 | 10:00 | 98.0% |
| A5 | 2/LD/T0/5 | 89 | ERS5329248 | 39687614 | 2020-08-24T09:00:00Z | 0 | 10:00 | 97.5% |
| A6 | 2/LD/T0/6 | 90 | ERS5329249 | 47388666 | 2020-08-24T09:00:00Z | 0 | 10:00 | 97.9% |
| A7 | 2/LD/T1/1 | 97 | ERS5329250 | 48544056 | 2020-08-24T13:00:00Z | 4 | 14:00 | 97.9% |
| A8 | 2/LD/T1/2 | 98 | ERS5329251 | 42958682 | 2020-08-24T13:00:00Z | 4 | 14:00 | 98.0% |
| A9 | 2/LD/T1/3 | 99 | ERS5329252 | 48892118 | 2020-08-24T13:00:00Z | 4 | 14:00 | 97.9% |
| A10 | 2/LD/T1/4 | 100 | ERS5329253 | 57346024 | 2020-08-24T13:00:00Z | 4 | 14:00 | 98.0% |
| A11 | 2/LD/T1/5 | 101 | ERS5329254 | 44973308 | 2020-08-24T13:00:00Z | 4 | 14:00 | 97.9% |
| A12 | 2/LD/T1/6 | 102 | ERS5329255 | 46567214 | 2020-08-24T13:00:00Z | 4 | 14:00 | 97.8% |
| A13 | 2/LD/T2/1 | 109 | ERS5329256 | 46177414 | 2020-08-24T17:00:00Z | 8 | 18:00 | 97.9% |
| A14 | 2/LD/T2/2 | 110 | ERS5329257 | 49718214 | 2020-08-24T17:00:00Z | 8 | 18:00 | 97.8% |
| A15 | 2/LD/T2/3 | 111 | ERS5329258 | 40568918 | 2020-08-24T17:00:00Z | 8 | 18:00 | 97.7% |
| A16 | 2/LD/T2/4 | 112 | ERS5329259 | 47615560 | 2020-08-24T17:00:00Z | 8 | 18:00 | 97.8% |
| A17 | 2/LD/T2/5 | 113 | ERS5329260 | 49860608 | 2020-08-24T17:00:00Z | 8 | 18:00 | 97.8% |
| A18 | 2/LD/T2/6 | 114 | ERS5329261 | 45925090 | 2020-08-24T17:00:00Z | 8 | 18:00 | 97.4% |
| A19 | 2/LD/T3/1 | 121 | ERS5329262 | 49124232 | 2020-08-24T21:00:00Z | 12 | 22:00 | 97.2% |
| A20 | 2/LD/T3/2 | 122 | ERS5329263 | 46414190 | 2020-08-24T21:00:00Z | 12 | 22:00 | 97.8% |
| A21 | 2/LD/T3/3 | 123 | ERS5329264 | 53764366 | 2020-08-24T21:00:00Z | 12 | 22:00 | 97.5% |
| A22 | 2/LD/T3/4 | 124 | ERS5329265 | 46872910 | 2020-08-24T21:00:00Z | 12 | 22:00 | 97.8% |
| A23 | 2/LD/T3/5 | 125 | ERS5329266 | 41175448 | 2020-08-24T21:00:00Z | 12 | 22:00 | 97.8% |
| A24 | 2/LD/T3/6 | 126 | ERS5329267 | 39772946 | 2020-08-24T21:00:00Z | 12 | 22:00 | 97.6% |
| A25 | 2/LD/T4/1 | 133 | ERS5329268 | 56203190 | 2020-08-25T01:00:00Z | 16 | 02:00 | 97.6% |
| A26 | 2/LD/T4/2 | 134 | ERS5329269 | 46268272 | 2020-08-25T01:00:00Z | 16 | 02:00 | 97.4% |
| A27 | 2/LD/T4/3 | 135 | ERS5329270 | 68576730 | 2020-08-25T01:00:00Z | 16 | 02:00 | 97.0% |
| A28 | 2/LD/T4/4 | 136 | ERS5329271 | 48524028 | 2020-08-25T01:00:00Z | 16 | 02:00 | 97.3% |
| A29 | 2/LD/T4/5 | 137 | ERS5329272 | 45795270 | 2020-08-25T01:00:00Z | 16 | 02:00 | 97.4% |
| A30 | 2/LD/T4/6 | 138 | ERS5329273 | 49615352 | 2020-08-25T01:00:00Z | 16 | 02:00 | 97.7% |
| A31 | 2/LD/T5/1 | 145 | ERS5329274 | 49813802 | 2020-08-25T05:00:00Z | 20 | 06:00 | 97.9% |
| A32 | 2/LD/T5/2 | 146 | ERS5329275 | 50785920 | 2020-08-25T05:00:00Z | 20 | 06:00 | 97.7% |
| A33 | 2/LD/T5/3 | 147 | ERS5329276 | 40505336 | 2020-08-25T05:00:00Z | 20 | 06:00 | 97.6% |
| A34 | 2/LD/T5/4 | 148 | ERS5329277 | 42291530 | 2020-08-25T05:00:00Z | 20 | 06:00 | 97.9% |
| A35 | 2/LD/T5/5 | 149 | ERS5329278 | 49107588 | 2020-08-25T05:00:00Z | 20 | 06:00 | 97.7% |
| A36 | 2/LD/T5/6 | 150 | ERS5329279 | 43759616 | 2020-08-25T05:00:00Z | 20 | 06:00 | 97.6% |
| A37 | 2/LD/T6/1 | 157 | ERS5329280 | 40172870 | 2020-08-25T09:00:00Z | 24 | 10:00+1 | 98.0% |
| A38 | 2/LD/T6/2 | 158 | ERS5329281 | 43202190 | 2020-08-25T09:00:00Z | 24 | 10:00+1 | 97.6% |
| A39 | 2/LD/T6/3 | 159 | ERS5329282 | 45730066 | 2020-08-25T09:00:00Z | 24 | 10:00+1 | 97.8% |
| A40 | 2/LD/T6/4 | 160 | ERS5329283 | 46641334 | 2020-08-25T09:00:00Z | 24 | 10:00+1 | 97.7% |
| A41 | 2/LD/T6/5 | 161 | ERS5329284 | 49706180 | 2020-08-25T09:00:00Z | 24 | 10:00+1 | 97.4% |
| A42 | 2/LD/T6/6 | 162 | ERS5329285 | 44532910 | 2020-08-25T09:00:00Z | 24 | 10:00+1 | 97.7% |

**Supplementary Table S2.** *S. salar* clock gene accessions and locations.

| Clock gene | Ensembl LOC_ID | NCBI Accession | NCBI Gene_ID | Chromosome location |
|---|---|---|---|---|
| *clock1a.1* | ENSSSAG00000056165 | XM_014169004.1 | LOC106584123 | ssa23:19987839-20018200 |
| *clock1a.2* | ENSSSAG00000003979 | XM_014122997.1 | LOC106560272 | ssa10:31345809-31398321 |
| *clock2a* | ENSSSAG00000072526 | XM_014137489.1 | LOC106567770 | ssa13:81971735-82044254 |
| *clock2b* | ENSSSAG00000063571 | XM_014196792.1 | LOC106603298 | ssa04:42639734-42675993 |
| *arntl1a.1* | ENSSSAG00000055510 | XM_014148046.1 | LOC106573213 | ssa16:12086304-12095636 |
| *arntl1a.2* | ENSSSAG00000063495 | XM_014125430.1 | LOC106561455 | ssa10:99088660-99104965 |
| *arntl1b.1* | ENSSSAG00000073101 | XM_014175326.1 | LOC106587199 | ssa26:18743473-18765117 |
| *arntl1b.2* | ENSSSAG00000068292 | XM_014126699.1 | LOC106562106 | ssa11:18402203-18411618 |
| *arntl2a* | ENSSSAG00000059155 | XM_014125375.1 | LOC106561420 | ssa10:94715917-94748435 |
| *arntl2b* | ENSSSAG00000008440 | XM_014153577.1 | LOC106576418 | ssa17:47744203-47759123 |
| *arntl2c* | ENSSSAG00000009920 | XM_014208404.1 | LOC106609509 | ssa07:45552387-45568717 |
| *per1a.1* | ENSSSAG00000077896 | XM_014196530.1 | LOC106603198 | ssa04:38522751-38532432 |
| *per1a.2* | ENSSSAG00000063164 | XM_014128896.1 | LOC106563389 | ssa11:73693186-73705207 |
| *per1b* | ENSSSAG00000002804 | XM_014206471.1 | LOC106608520 | ssa07:4731276-4739498 |
| *per2a* | ENSSSAG00000076981 | XM_014139595.1 | LOC100301980 | ssa14:16742620-16769326 |
| *per2b* | ENSSSAG00000001704 | XM_014190838.1 | LOC106599551 | ssa03:17486279-17517618 |
| *per3* | ENSSSAG00000077674 | XM_014166967.1 | LOC106583133 | ssa22:25358497-25382546 |
| *cry1a.1* | ENSSSAG00000077590 | XM_014148221.1 | LOC106573292 | ssa16:15906400-15976136 |
| *cry1a.2* | ENSSSAG00000071693 | XM_014125353.1 | LOC106561407 | ssa10:95086446-95106453 |
| *cry2* | ENSSSAG00000073888 | XM_014125728.1 | LOC106561609 | ssa10:105488616-105553942 |
| *cry3b.1* | ENSSSAG00000081201 | XM_014131850.1 | LOC106565112 | ssa12:46213293-46226192 |
| *cry3b.2* | ENSSSAG00000081121 | XM_014167724.1 | LOC106583488 | ssa22:40956787-40966869 |
| *cry4* | ENSSSAG00000069768 | XM_014176427.1 | LOC106587781 | ssa26:36623393-36647646 |
| *cry5* | ENSSSAG00000079794 | XM_014138346.1 | LOC106568206 | ssa13:86762607-86774549 |
| *nr1d1a* | ENSSSAG00000003153 | XM_014204497.1 | LOC106607496 | ssa06:40433770-40444554 |
| *nr1d1b* | ENSSSAG00000007825 | XM_014192527.1 | LOC106600825 | ssa03:57681841-57689097 |
| *nr1d2a.1* | ENSSSAG00000037545 | XM_014164862.1 | LOC106582110 | ssa02:25864163-25873212 |
| *nr1d2a.2* | ENSSSAG00000005012 | XM_014200712.1 | LOC100136378 | ssa05:54986045-54995902 |
| *nr1d2b.1* | ENSSSAG00000056065 | XM_014142438.1 | LOC106570286 | ssa14:68249965-68261020 |
| *nr1d2b.2* | ENSSSAG00000054663 | XM_014177878.1 | LOC106588668 | ssa27:19843632-19851666 |
| *nr1d4a.1* | ENSSSAG00000065806 | XM_014147027.1 | LOC106572653 | ssa15:88787769-88813331 |
| *nr1d4a.2* | ENSSSAG00000077410 | XM_014135912.1 | LOC106567064 | ssa13:35389895-35404997 |
| *nr1d4b.1* | ENSSSAG00000081117 | XM_014167340.1 | LOC106583303 | ssa22:32346247-32358789 |
| *nr1d4b.2* | ENSSSAG00000067342 | XM_014132672.1 | LOC106565463 | ssa12:54465885-54474575 |

**Supplementary Table S2.** *Cont. S. salar* clock gene accessions and locations.

| Clock gene | Ensembl LOC_ID | NCBI Accession | NCBI Gene_ID | Chromosome location |
|---|---|---|---|---|
| *roraa.1* | ENSSSAG00000066827 | XM_014148628.1 | LOC106573506 | ssa16:27588967-27706002 |
| *roraa.2* | ENSSSAG00000074849 | XM_014124804.1 | LOC106561150 | ssa10:82919138-82990987 |
| *rorb.1* | ENSSSAG00000069458 | XM_014160683.1 | LOC106580068 | ssa20:14747696-14776762 |
| *rorb.2* | ENSSSAG00000065430 | XM_014172220.1 | LOC106585703 | ssa24:30374236-30414448 |
| *rorca.1* | ENSSSAG00000073620 | XM_014169656.1 | LOC106584392 | ssa23:30486289-30543797 |
| *rorca.2* | ENSSSAG00000068177 | XM_014216453.1 | LOC106613816 | ssa10:21617008-21834653 |
| *rorcb.1* | ENSSSAG00000079594 | XM_014140953.1 | LOC106569518 | ssa14:38121993-38144650 |
| *rorcb.2* | ENSSSAG00000080721 | XM_014191359.1 | LOC106600026 | ssa03:37981172-38001032 |
| *csnk1db.1* | ENSSSAG00000068205 | XM_014179815.1 | LOC106589616 | ssa28:16447101-16461477 |
| *csnk1db.2* | ENSSSAG00000003495 | XM_014209485.1 | LOC106610228 | ssa01:77662396-77680525 |
| *csnk1ea* | ENSSSAG00000001530 | XM_014193266.1 | LOC106601228 | ssa03:65302128-65311565 |
| *csnk1eb* | ENSSSAG00000068400 | NM_001140384.1 | LOC100195355 | ssa06:33906202-33916152 |
| *csnk1ec* | ENSSSAG00000015336 | XM_014179651.1 | LOC106589542 | ssa28:14144088-14151122 |
| *csnk1ed* | ENSSSAG00000003438 | XM_014208340.1 | LOC106609470 | ssa01:74533732-74548069 |

**Supplementary Table S3.** Number of significant genes: Rhythmic (RAIN) or circadian (JTK) depending on the P-value (FDR adjusted) or relative Amplitude thresholds used. All dataset and clock genes only (in bracket).

| | RAIN | JTK | overlap |
|---|---|---|---|
| adjP-value $< 0.05$ | | | |
| rAmp $\geq 0$ | 12,322 (32) | 6,485 (22) | 6,427 (22) |
| rAmp $\geq 5\%$ | 9,818 (25) | 5,954 (21) | 5,896 (21) |
| rAmp $\geq 10\%$ | 5,529 (20) | 3,678 (18) | 3,621 (18) |
| rAmp $\geq 15\%$ | 2,906 (8) | 2,007 (8) | 1,959 (8) |
| adjP-value $< 0.01$ | | | |
| rAmp $\geq 0$ | 9,064 (27) | 3,786 (15) | 3,760 (15) |
| rAmp $\geq 5\%$ | 7,413 (24) | 3,627 (15) | 3,602 (15) |
| rAmp $\geq 10\%$ | 4,234 (19) | 2,374 (14) | 2,349 (14) |
| rAmp $\geq 15\%$ | 2,206 (7) | 1,264 (6) | 1,241 (6) |
| adjP-value $< 0.001$ | | | |
| rAmp $\geq 0$ | 5,815 (22) | 1,721 (11) | 1,717 (11) |
| rAmp $\geq 5\%$ | 4,911 (21) | 1,702 (11) | 1,698 (11) |
| rAmp $\geq 10\%$ | **2,864 (16)** | **1,215 (11)** | **1,211 (11)** |
| rAmp $\geq 15\%$ | 1,470 (6) | 648 (5) | 644 (5) |
